# Unveiling the Challenges in Tandem Ureteral Stent Management for Malignant Ureteral Obstruction: Failure Rate, Risk Factors, and Durability of Their Replacement

**DOI:** 10.3390/jcm12165251

**Published:** 2023-08-11

**Authors:** Orel Carmona, Asaf Shvero, Dorit E. Zilberman, Zohar A. Dotan, Nir Kleinmann

**Affiliations:** 1 The Department of Urology, Sheba Medical Center, Ramat Gan 5262000, Israelnir.kleinmann@sheba.health.gov.il (N.K.); 2 School of Medicine, Tel Aviv University, Tel Aviv 6997801, Israel

**Keywords:** tandem ureteral stents, malignant ureteral obstruction, polymeric stents, ureteral stricture, renal drainage

## Abstract

Background: Malignant ureteral obstruction (MUO) is a sequela of advanced malignant disease that requires renal drainage, with tandem ureteral stents (TUSs) being a viable option. This study aimed to evaluate the TUS failure rate, associated risk factors, and the feasibility of replacing failed TUSs with a new pair of stents. Methods: A retrospective analysis of MUO patients treated with TUS insertion from 2014 to 2022 was conducted. TUS failure was defined as urosepsis, recurrent urinary tract infections, acute kidney failure, or new hydronephrosis on imaging. Cox proportional hazard regression analysis identified the independent predictors of TUS failure. Results: A total of 240 procedures were performed on 186 patients, with TUS drainage failing in 67 patients (36%). The median time to failure was 7 months. Multivariate analysis revealed female gender (OR = 3.46, *p* = 0.002), pelvic mass (OR = 1.75, *p* = 0.001), and distal ureteral obstruction (OR = 2.27, *p* = 0.04) as significant risk factors for TUS failure. Of the failure group, 42 patients (22.6%) underwent TUS replacement for a new pair. Yet, 24 (57.2%) experienced a second failure, with a median time of 4.5 months. The risk factors for TUS second failure included a stricture longer than 30 mm (OR = 11.8, *p* = 0.04), replacement with TUSs of the same diameter (OR = 43, *p* = 0.003), and initial TUS failure within 6 months (OR = 19.2, *p* = 0.006). Conclusions: TUS insertion for the treatment of MUO is feasible and has good outcomes with a relatively low failure rate. Primary pelvic mass and distal ureteral obstruction pose higher risks for TUS failure. Replacing failed TUSs with a new pair has a success rate of 42.8%. Consideration should be given to placing larger diameter stents when replacing failed TUS.

## 1. Introduction

Malignant ureteral obstruction (MUO) is a urological sequela of advanced malignant diseases, most of which are not urologic malignancies [1,2]—mostly cervical, ovarian, and colorectal cancers [3,4]. This urologic condition may present with complications such as flank pain, acute kidney injury, hydronephrosis, recurrent urinary tract infections, and urosepsis [1,5]. The obstruction may be the result of intrinsic ureteral obstruction or extrinsic ureteral compression due to the primary tumor, lymphadenopathy, or metastasis [6]. A definitive treatment may be a surgical reconstruction of the ureter, but given the short life expectancy of this population of patients [7,8] and their frailty, there is a shift toward minimally invasive techniques, including percutaneous nephrostomy tube (PCN) insertion, single or tandem double-pigtail ureteral stents, and metallic ureteral stents [3,9,10].

Drainage of the kidney with ureteral stents might fail due to several reasons: migration of the stents (proximally or distally), obstruction of the stent lumen with mucous production from the irritated urothelium or urothelial sloughing, encrustation, and blockage of the stents (usually at their distal curl), or progressive worsening of the external pressure from disease progression or fibrotic tissue [11,12].

A single ureteral stent has a failure rate of about 40% [3], with most patients developing complications [3,13]. Tandem ureteral stents (TUSs)—the use of two individual double-pigtail stents placed side-by-side—have been shown to be efficient with a success rate of 73–85% [14,15] due to their ability to withstand higher external pressure, enable urine flow in the space between the two stents in addition to the flow through the stents [16,17], and the fact they serve as a backup for one another in case one fails [18,19].

Currently, there are no specific guidelines regarding the treatment of MUO, let alone the treatment options for patients who had TUSs that failed in order to drain the kidney.

The aim of this study is to analyze the risk factors for the failure of TUS drainage in cases of MUO and assess the feasibility of replacing a failed pair with a new pair of stents.

## 2. Materials and Methods

### 2.1. Study Population

After receiving institutional review board approval, we reviewed the medical records of all patients who underwent TUS insertion in our institution due to MUO between 2014 and 2022.

Patients who present with malignant ureteral obstruction to the outpatient clinic are referred directly to either TUS insertion or PCN insertion, after a shared decision-making discussion with each patient. At our institution, we do not offer single stent drainage to patients with MUO due to the higher failure rate of a single stent compared to tandem stents [4,13,14,15]. Patients who present to our emergency department with urosepsis, acute kidney injury, or anuria due to MUO are drained via a PCN urgently.

We collected the following data: age, gender, location and type of the primary tumor, previous treatments including surgery, radiotherapy, chemotherapy, and immunotherapy, side of the obstruction, obstruction site and length as measured by retrograde ureterography, ureteral stent diameter and length, serum creatinine levels and hydronephrosis level prior to and after TUS insertion, number of TUS replacements, time to stent failure, failure reason, and further treatment.

The follow-up protocol included office visits with renal ultrasonography, urinalysis, urine culture, CBC, and SMAC. Visits were scheduled at 1 month after stent insertion and every 2 months thereafter until the elective time for stent exchange was scheduled (usually 12 months after the previous exchange/insertion).

Stent failure was defined as an acute rise in creatinine level, worsening hydronephrosis per imaging tests, and recurrent urinary tract infections leading to hospitalizations or urosepsis. Lastly, the date and reason for patient demise were documented.

### 2.2. Surgical Technique

All procedures were performed by four endourological fellowship-trained surgeons. With the patients under general anesthesia, and in dorsal lithotomy position, a semi-rigid ureteroscope was inserted into the bladder and the ureteral orifices were identified. A 10 Fr dual-lumen catheter was inserted into the ureters over a guidewire and a retrograde ureteropyelography was performed in order to define the location and length of the obstruction. In cases in which a 10 Fr dual-lumen catheter could not pass beyond the ureteral orifice due to stricture, ureteral dilation was performed, either by serial ureteral dilators or by a 4 cm/18F ureteral balloon catheter filled until reaching a pressure of 20 atmospheres. In cases of a long stricture or multiple strictures, several dilations were performed. Then the 10 Fr dual-lumen catheter was reinserted, a second guidewire was passed into the kidney, and two double-pigtail stiff Percuflex™ ureteral stents (Boston Scientific, Marlborough, MA, USA) were placed simultaneously over the guidewires under fluoroscopy. The diameter and length of the stents were decided by the surgeon; the sizes available at our institute are either 6 or 7 Fr, with lengths of 22–30 cm. At our institution, the selected stents are usually two stents of 6 Fr each in the first insertion of TUSs. After failure, the diameter selection was at the surgeons’ discretion, usually to either 6 + 7 Fr or 7 + 7 Fr.

The proximal curls were positioned within an upper calyx or the renal pelvis, and the distal curls were formed in the bladder.

### 2.3. Statistical Analysis

Statistical analyses were performed with IBM SPSS software, version 26.0 (Armonk, NY, USA: IBM Corp, 2019). We used continuous variables to describe the median (interquartile range [IQR]) and frequency (proportions) to describe the categorical variables. Cox proportional hazard regression analysis was used to assess the independent predictors of time to TUS failure. Statistically significant predictors on univariate analysis were included in the multivariate analysis. Statistical significance was defined as *p* < 0.05.

## 3. Results

**Figure 1:**
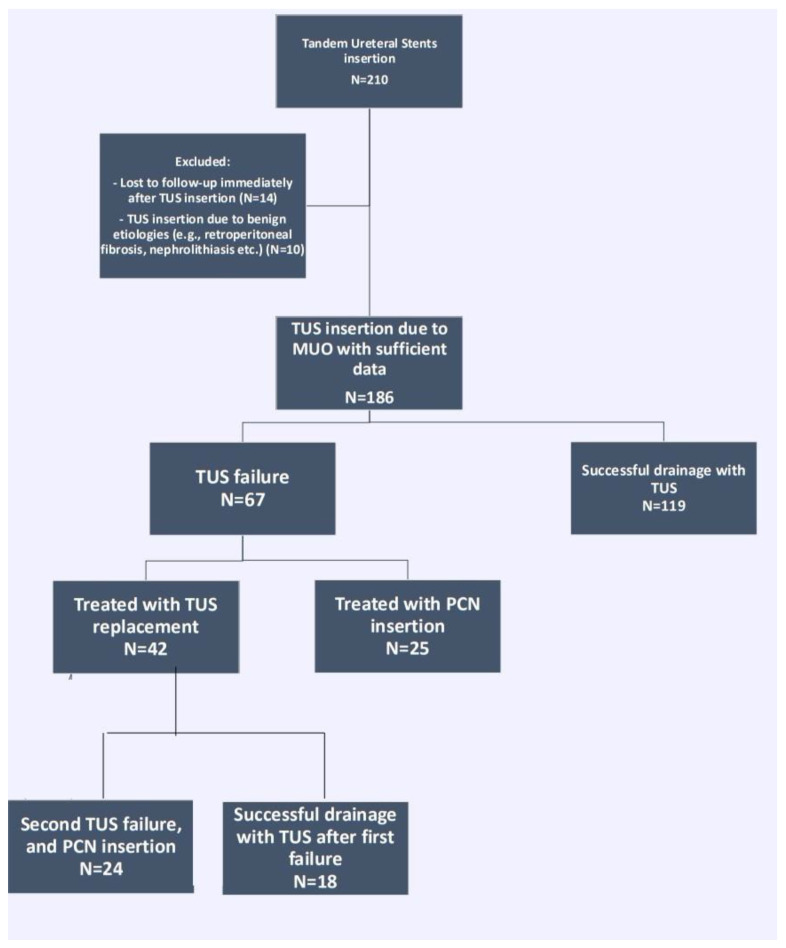
Flowchart of patient identification and study groups. TUS = tandem ureteral stents insertion, MUO = malignant ureteral obstruction, PCN = percutaneous nephrostomy tube.

A total of 186 patients underwent TUS insertion to 240 renal units during the study period. A flowchart of patients’ inclusion and exclusion is represented in Figure 1. Patient and stricture characteristics are shown in Table 1.

### 3.1. The Successful TUS Drainage Group

During a median follow-up time of 14 months (IQR 6–25.5), the drainage of 119 patients with TUSs (64%) was successful, and they continued to undergo timely replacements of the stents yearly. This group underwent a total of 268 TUS replacements, with a mean of 2.25 ± 1.67 per patient. During the follow-up time, 55 patients out of this group (46.2%) died, in a median time of 7 months (IQR 3–15).

### 3.2. The Failed TUS Group

In 67 patients (36%), the drainage failed during the follow-up period. The median time to failure was 7 months (IQR 4–17). In total, 18 (27%) patients experienced sepsis, 3 (4.5%) patients suffered from culture-proven recurrent urinary tract infections leading to recurrent hospitalizations, 33 (49.2%) patients had a new finding of hydronephrosis after prior imaging had shown resolution of the hydronephrosis after TUS insertion, and 8 (11.9%) patients had acute kidney injury.

During the follow-up time, 28 patients (41.8%) out of this group died, in a median time of 16 months (IQR 8.25–21.25).

Of the 67 patients in whom the drainage failed, 24 (35.8%) experienced failure after the first TUS insertion, 27 (40.3%) after one elective replacement of the stent, 10 (14.9%) after two elective replacements, 3 (4.5%) after three elective replacements, and 1 per group after four (1.5%), five (1.5%), and eight (1.5%) elective replacements. The median number of elective TUS replacements before the first failure was one procedure (IQR 0–1). In total, the failure group underwent 172 procedures for TUS replacement, with a mean and standard deviation of 2.5 ± 1.7 procedures per patient.

### 3.3. Oncological Background

**Figure 2:**
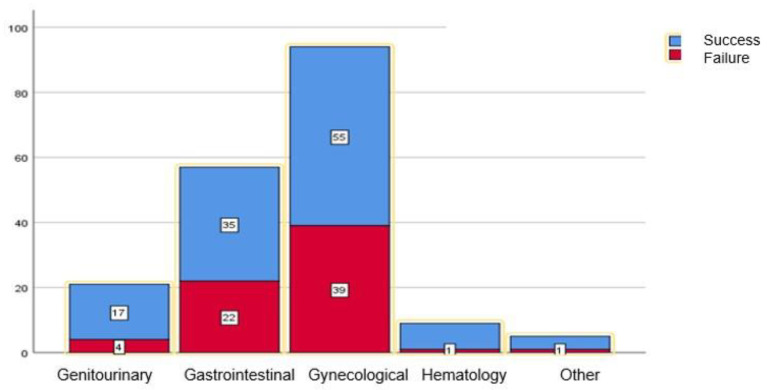
Proportion of tandem ureteral stent drainage success (blue) and failure (red), categorized by primary tumor system. The number of patients is indicated in each box. There were higher rates of failure in gynecological (41.4%) and gastrointestinal (38.6%) malignancies, in comparison to urological (19%), hematological (11%) and others (20%). However, this did not reach statistical significance (*p* = 0.136).

The risk of failure was 41.4% in the gynecological malignancies, 38.6% in the gastrointestinal malignancies, 19% in the genitourinary malignancies, and 11% in the hematological malignancies (Figure 2). These proportions of the primary tumor origin were not different between the patients in whom the TUSs failed and those in whom they succeeded (*p* = 0.137). 

When highlighting the most common primary tumor locations per system, failure occurred in 19 of the 38 cervical cancer patients (50%); in 14 of the 32 ovarian cancer patients (44%); in 11 of the 23 rectal cancer patients (47.8%); and in 8 of the 23 colon cancer patients (34.7%). TUSs were most successful in prostate cancer patients, where only one out of 10 (10%) patients had his TUSs fail.

### 3.4. Risk Factors for TUS First Failure

When comparing the group with failed TUSs to the group with successful procedures, univariate and multivariate analyses showed that being of female gender (OR = 3.46, *p* = 0.002), having a pelvic mass as the primary tumor (OR = 1.75, *p* = 0.001), and the presence of distal ureteral obstruction (OR = 2.27, *p* = 0.04) are risk factors for TUS failure. Pelvic radiation therapy, stricture length, and the subtype of the primary tumor were all included in the analyses but were not found to be a statistically significant risk factor for TUS failure.

**Figure 3:**
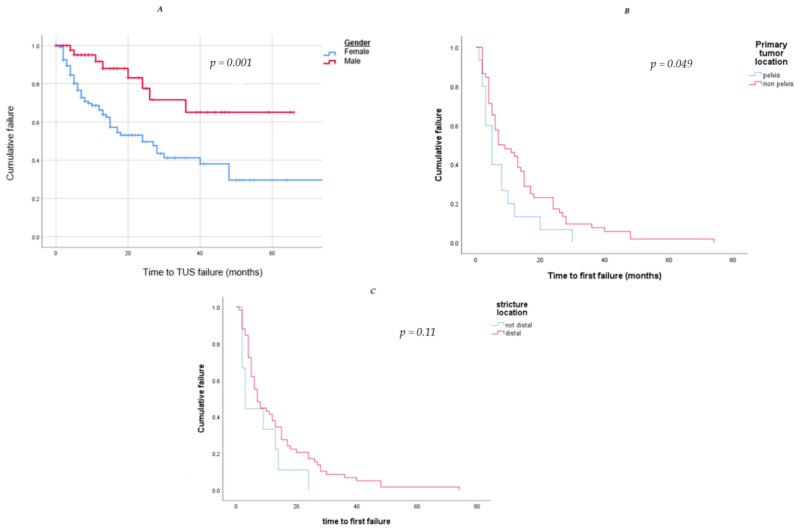
Kaplan–Meier curves to estimate the success of TUS drainage by (**A**). Gender (blue—female, red—male); we found a significant success rate in the male group in comparison to the female group (*p* = 0.001). (**B**). Primary tumor location (blue—pelvic, red—not-pelvic); we found a significant success rate in the non-pelvic tumor group in comparison to the pelvic tumor group (*p* = 0.049). (**C**) Structure location (red—distal, blue—not-distal); although distal structure was found to be a significant risk factor for TUS failure, the survival analysis did not show a higher success rate in the non-distal structure group (*p* = 0.11).

In a Kaplan–Meier curve to estimate the success of TUS drainage by gender, we found a significant success rate in the male group in comparison to the female group (*p* = 0.001). (Figure 3). The mean time to failure in the female group was 12.1 ± 13.7 months, in comparison to 17.4 ± 11.1 months in the male group.

### 3.5. Treatment after First TUS Drainage Failure

In total, 25 (13.4%) out of the 67 patients with failed TUSs underwent a PCN insertion, while 42 (22.6%) patients were treated by replacing the TUSs with a new pair of TUSs. Those who were treated by replacing the TUSs with a new pair of stents subsequently underwent a median of two elective changes of the TUSs (IQR 1–4).

### 3.6. Outcomes of Replacing TUSs with a New Pair after One Failure

Of the 42 patients whose TUSs were replaced with a new pair of stents after the first failure, during a median follow-up time of 20 months (14–52), 18 patients (42.8%) continued to experience successful drainage with the TUSs and underwent periodical replacements. On the other hand, 24 patients (57.2%) experienced a second failure, in a median follow-up time of 4.5 months (2–7.25). All of these patients were subsequently drained with a PCN.

Overall, the median time to the next TUS replacement, out of the 42 patients who were treated with TUS replacement for the first failure, was 7.5 months (IQR 4–12). Yet, when analyzing separately those who experienced failure for a second time and those who did not, the median time to the next TUS replacement was 5.5 months (IQR 2.25–7.75) vs. 10.5 months (IQR 8.75–12), respectively.

**Figure 4:**
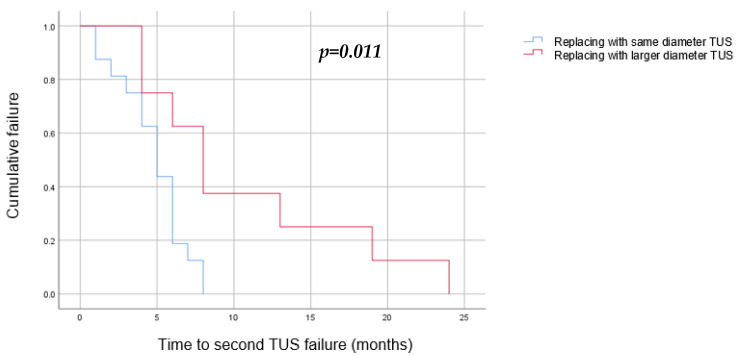
Kaplan–Meier curve to estimate the success of TUS replacement after a single failure by TUS diameter (blue—same diameter as the TUS that have failed, red—larger diameter). We found a significant success rate when replacing the TUS with a larger-diameter stent (*p* = 0.011).

**Figure 5:**
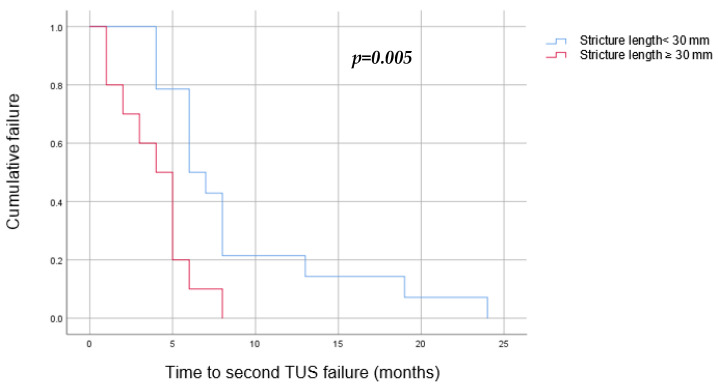
Kaplan–Meier curve to estimate the success of TUS replacement after a single failure by stricture length (blue—stricture shorter than 30 mm, red—stricture longer than 30 mm). We found that a stricture longer than 30 mm was related to an earlier second failure, with a median of 4 months (2–5), in comparison to 6 months (5–8) in those with shorter strictures.

Univariate and multivariate analyses have found the following to be risk factors for a second TUS failure: stricture length longer than 30 mm (OR = 11.8, *p* = 0.04), replacing the failed TUSs with TUSs of the same diameter (OR = 43, *p* = 0.003), and early first failure, defined as a failure during the first 6 months of the first TUS insertion (OR = 19.2, *p* = 0.006) (Table 2). A Kaplan–Meier survival estimate was computed to assess the time until TUS failure in two groups of patients: those whose TUSs were replaced with a larger diameter pair, and those whose TUSs were replaced with a same diameter pair. We found that replacing the TUSs with larger diameter ones elongated the time to a second failure, from a median of 5 months (3–6) when using the same diameter stents to 8 months (4–13) (*p* = 0.011) (Figure 4). In addition, we found that a stricture longer than 30 mm was related to an earlier second failure, with a median of 4 months (2–5) in comparison to 6 months (5–8) in those with shorter strictures (*p* = 0.05) (Figure 5).

## 4. Discussion

MUO is a rare sign of advanced malignant disease and can result from extrinsic compression by a primary lesion, metastasis, retroperitoneal or pelvic lymphadenopathy, or direct tumor seeding [3,6]. While reconstructive strategies exist, the grim prognosis of the majority of these patients, alongside their frail state, motivates treatment in a less invasive manner. Due to the high failure rate of single ureteral stent insertion, other approaches have been pursued, such as tandem ureteral stent insertion [3,14,20].

A prospective study by Liu et al. [18] demonstrated the longer stent patency of TUSs compared to single ureteral stent drainage, with a similar complication rate. Of note, in their study, was that all ureteral stenting procedures were performed with an antegrade approach, unlike our approach.

In our study, we found that it is feasible to replace failed TUSs with a new pair of TUSs, with a success rate of 42.8%.

***Stricture length***. We demonstrated that patients with strictures of 30 mm and longer have a higher risk for a second TUS failure, with a shorter time to a second failure in comparison to patients with strictures shorter than 30 mm (a median of 4 versus 6 months respectively, *p* = 0.05). Previously published literature regarding both benign and malignant strictures has discussed the relationship between the success rate of endoscopic treatment and the stricture’s length.

Lu et al. [21] published a meta-analysis regarding the endoscopic treatment of ureteral strictures. Although their meta-analysis included patients with benign ureteral strictures exclusively, they found, similarly to our findings, that endoscopic treatment of strictures shorter than 20 mm achieved better success rates (69% vs. 19%, OR = 0.13). Reus et al. [22] published their experience of treating post-malignancy ureteral strictures endoscopically, via balloon dilation or dilatation catheters, and found a 20% recurrence rate within this group of patients, 80% of them with strictures longer than 20 mm.

A risk stratification score for MUO was published by Izumi et al. [23] based on primary cancer site, laterality, serum creatinine, and treatment for primary cancer. Longer median stricture length was found in the poor risk group compared to the intermediate and good risk groups (32 mm in comparison to 28 and 24 mm, respectively, *p* = 0.0045). This may serve as an explanation for the higher risk of a second TUS failure in our study, although we did not use the PLaCT risk grouping on our cohort.

***Time to first failure***. We found that when the first TUS failure was shortly after its original insertion (less than 6 months—“early”), patients had 11.8 times the risk of a second failure compared to when the failure occurred later than 6 months (“late”). In a subgroup analysis of those whose TUSs failed early, compared to those whose TUSs failed late, more patients had locally advanced disease prior to the first TUS insertion (54.3% vs. 26.7%, OR = 3.26, *p* = 0.043). Other parameters of a more advanced oncological primary disease, such as metastatic disease prior to the first TUS insertion (77.5% vs. 60%, OR = 2.2, *p* = 0.186), the number of surgeries prior to the TUS insertion (mean of 1.23 ± 0.8 vs. 0.97 ± 0.8, *p* = 0.218), and the development of metastatic disease after the first TUS insertion and before the first failure (71.4% vs. 43%, OR = 3.3, *p* = 0.361), were not found to be significant risk factors. This is in contrast to Cordeiro’s prognostic model [24], which indicates a relationship between survival and events related to malignant dissemination in patients with MUO drained by either a PCN or ureteral stenting. An external validation of this model was recently published [25], on a cohort of patients drained solely with TUS, which further validated Cordeiro’s finding in this particular group.

These data reflect that patients with long strictures who experienced failure early after the first TUS insertion will probably benefit from PCN insertion rather than TUS replacement, and these factors should be taken into consideration when consulting a patient with MUO after a singular TUS failure.

***Intra-operative parameters***. Our data indicate that the most significant risk factor for a second failure was replacing the TUSs with the same diameter stents (OR 43). These TUSs also failed earlier for the second time, in comparison to those whose TUSs were replaced with a larger diameter (5 months vs. 8 months, *p* = 0.011). According to our findings, it is of the utmost importance that when the treatment strategy for TUS failure is a replacement for a new pair of stents, they should be replaced with a larger diameter pair. A study by Rosen et al. [26] has previously demonstrated a reduced likelihood of reaching stent occlusion with either larger diameter stents or tandem stents compared to a single stent. According to their proposed mechanism, a greater surface area needs to be obstructed to cause significant stent blockage and hence failure.

As part of our department technique, before replacing a failed pair of TUSs with a new pair, we redilated the strictures with an endoscopic balloon, a 10 Fr dual-lumen catheter, or serial ureteral dilators. Reus et al. [22] demonstrated a 33% success rate of the redilation of strictures after one dilation failure. However, their study included both benign and malignant ureteral strictures, and they left a single 6 Fr Percuflex™ stent for 6 weeks. We believe that our higher retreatment success rate of 42.8%, especially with the current population of malignant obstruction, is partially related to the continuous stent drainage of tandem ureteral stents with a larger cumulative diameter.

The concept of balloon dilations of ureteral strictures is not new, for both malignant [1,27] and benign [21,28] strictures. Hu et al. [29] created a nomogram to predict stricture-free survival in patients with ureteral stricture after balloon dilation, and found that malignant stricture, chronic kidney disease, urinary nitrite, stent retention time, and balloon size were independent risk factors for the relapse of the ureteric stricture. Of note is that their cohort excluded patients with active malignant disease. To our knowledge, no external validation of this tool was made on patients with ureteric stricture secondary to active, advanced metastatic disease.

***Risk factors for 1st TUS failure***. The failure rate of TUSs in our study was 36%, which is higher than some of the previously described series in the literature. Liu et al. [18] reported a TUS failure rate of 5%; however they replaced the TUSs electively every 6 months. Varnavas et al. [30] reported a failure rate of 20%; however they did not specify the replacement interval and their cohort included only 15 patients.

At our institution, we use Percuflex® ureteral stents (Boston Scientific, Marlborough, MA, USA) and replace them every 12 months—the upper limit specified by the manufacturer. This reduces the number of elective admissions, number of procedures, and total duration of anesthesia. It is possible that this long interval is responsible for the relatively high failure rate.

Another factor that should be considered when exploring the failure mechanism is stent stiffness. A study by Vogt et al. [31] has previously demonstrated a decrease in the stent failure rate when using specific stiff reinforced stents. Remarkably, their findings revealed that only 11.1% of patients experienced stent failure when using 8F reinforced tandem stents. The question of whether the difference in the failure rate is attributed to the stent stiffness or the larger diameter of the tandem stents should be further explored in a prospective, randomized trial.

The median time to TUS failure was 7 months, which is slightly longer than other series published recently, which reported a 4-month median time to failure [31,32]. Median survival after TUS failure was 8 months, and median follow-up time was 17 months—which is longer than previous series [3,30], yet similar to more recent publications [31,33]. We believe that this indicates the improvement of systemic oncologic treatments for different malignancies in recent years, partially due to emerging novel immunotherapies, prolonging these patients’ life expectancy, and furthermore emphasizing the importance of effective urinary drainage for longer intervals.

We found that female gender, pelvic mass, and distal ureteral obstruction were associated with an increased risk of TUS failure. Previous studies have established that distal ureteral strictures present a noteworthy risk factor for ureteral stent failure [34,35]. The distal ureter appears to be particularly vulnerable to iatrogenic ischemic injury during gynecological or sigmorectal interventions and to non-ischemic damage from radiotherapy as part of the malignancy treatment in gynecological, urological, and surgical malignancies, as well as to direct invasion of the tumor to the ureter or complete encapsulation of the ureter in patients with pelvic masses. We believe that the three interconnected risk factors, namely distal ureteral obstruction, pelvic mass, and female gender, are associated with more severe, advanced gynecological or sigmorectal malignancies, thus explaining the increased risk of TUS failure. These results emphasize the challenge we have when consulting patients with gynecological and rectal malignancies, which, as shown previously, are the majority of MUO patients. More frequent office follow-up sessions and TUS exchanges should be considered in this group of patients, as well as different treatment strategies such as PCN or primary TUS insertion with larger diameter stents (6 Fr + 7 Fr or 7 Fr + 7 Fr). This relatively high failure rate must be communicated to patients before a decision is made to treat with TUSs.

This study provides practical data to be considered when consulting patients with MUO, when considering primary TUS or PCN insertion, and for those whose TUS insertions failed. These risk factors should be considered when counselling patients regarding the optimal treatment strategy, with every approach having its pros and cons [36].

One of the major advantages of TUSs is their lack of external devices. When comparing patients drained with PCNs versus ureteral stents due to acute ureteral obstruction secondary to stone disease, the PCN group had worse symptoms related to mobility, decline in usual activity, and personal hygiene, whereas the DJS group had more urinary-related symptoms [37]. Since patients with MUO usually require life-long drainage, factors such as personal hygiene, mobility, and the ability to continue with daily activity are of the outmost importance.

A previous study published by our group [36] has assessed the quality of life of MUO patients drained with either TUSs or PCNs, using the European Quality of Life Five Dimension Five Level Scale (EQ-5D-5L) and the “tube symptoms” questionnaire. No significant difference was found between the two groups in any EQ-5D-5L or tube symptom scores. However, in a subgroup analysis of the patients who experienced both methods of drainage, 84% reported that they preferred TUS over PCN drainage.

Our study included patients with MUO drained via either PCNs or TUSs. However, several other drainage and diversion methods for the urinary system exist. For example, metallic ureteral stents provide a higher resistance to external compression, and a higher stiffness compared with non-metallic stents [38,39]. A recent study found no significant difference in the mean success rate between several types of stents—Resonance® (Cook Urological, Bloomington, IN, USA), Memokath^®^ 051 (PNN Medical A/S, Kvistgård, Denmark), Allium (Allium medical solutions, Caesarea, Israel), and Uventa (Taewoong Medical, Seoul, Republic of Korea) all demonstrated a mean success rate of 60–74.5% [40].

As previously mentioned, reinforced stents are available, with their internal layer reinforced for extra resistance to compression [31]. The Detour extra-anatomic stent (Coloplast, Denmark) provides a permanent bypass of complete ureteral obstruction, by bypassing the obstruction and connecting the renal pelvis directly to the bladder [41], with a 66.7% success rate [40].

Ureteral strictures can be effectively treated through endoureterotomy using a cold knife, electrosurgical probe, or laser fiber. The available data primarily focus on benign strictures and demonstrate a decent success rate when combined with stent placement [42,43,44].

Surgical repair, using the open, laparoscopic or robotic approaches, remains a viable option for those MUO patients with a good prognosis and stable malignant disease. Depending on the location and length of the obstructed segment, uretero-ureterostomy or uretero-neocystostomy with or without a psoas hitch or boari flap can be performed. In situations where a long segment is obstructed, trans-ureteroureterostomy, ileal ureter, or renal auto-transplantation are appropriate alternatives [45,46,47].

To our knowledge, our study is the largest regarding TUS drainage in patients with MUO. Yet, it is not without limitations. First, due to its retrospective nature, there is a risk of selection bias, as management decisions are guided by physician subjective assessment and preference, which may affect first and second failure rates as well as drainage selection after the first failure. Second, we did not include quality-of-life data, which is important in this specific group of patients. Third, in this research, we used Percuflex™ stents exclusively, which are non-reinforced stents, and in all cases we inserted tandem stents without first attempting drainage with a single stent. Finally, this study comprises of a relatively small number of patients due to the rarity of this condition, hence finding prognostic factors is relatively difficult.

## 5. Conclusions

Our study shows that tandem ureteral stent insertion for the treatment of MUO is feasible and has good outcomes with a relatively low failure rate. Patients at the highest risk of TUS failure are those with a primary pelvic mass and distal ureteral obstruction. Replacing the failed stents with a new pair of TUSs is a feasible strategy with an acceptable success rate. The treatment plan should be tailored for each MUO patient, with their unique disease and stricture characteristics.

## Figures and Tables

**Table 1:** Patients and stricture characteristics.

Number of patients	186
**Number of renal units**	240 (54 bilateral TUS)
**Age (years), median (IQR)**	65 (52–74)
**Gender (female), n (%)**	140 (75.3%)
**Primary tumor site, n (%)**	
**Gynecologic**	94 (50.5%)
**Gastrointestinal**	57 (30.6%)
**Genitourinary**	21 (11.3%)
**Hematology**	9 (4.8%)
**Other**	5 (2.7%)
**Metastasis at presentation, n (%)**	141 (75.8%)
**Previous radiation therapy, n (%)**	
**Pelvic**	86 (46.2%)
**Retroperitoneal**	6 (3.2%)
**None**	94 (50.5%)
**Serum Creatinine before drainage, median (IQR)**	1.3 (0.89–1.72)
**Prior nephrostomy tube, n (%)**	33 (17.7%)
**Side of stricture, n (%)**	
**Right**	65 (34.9%)
**Left**	67 (36%)
**Bilateral**	54 (29%)
**Stricture length, mm, median (IQR)**	20 (10–30)
**Stricture location, n (%)**	
**Proximal**	7 (3.8%)
**Medial**	33 (17.7%)
**Distal**	146 (78.5%)

**Table 2:** Risk Factors of second Tandem Stent Drainage Failure.

			Univariate Analysis			*Multivariate Analysis*		
Risk Factor	Success n = 18	Failure n = 24	OR	95% CI	p	OR	95% CI	p
Age, years, median (IQR)	64 (54, 66.5)	56.6 (50, 71)	0.991	0.94, 1.04	0.736			
Gender, Female, n (%)	16 (88.9%)	18 (75%)	0.375	0.06, 2.12	0.268			
Weight, kg, median (IQR)	63 (53, 74)	63 (55, 68)	0.999	0.96, 1.03	0.961			
Primary tumor system, n, (%)								
*Gynecological*	12 (66.6%)	10 (41.7%)	0.83	0.08, 2.1	0.670			
*Gastrointestinal*	5 (27.8%)	11 (45.8%)	0.379	0.09, 1.4	0.159			
*Genitourinary*	1 (5.6%)	3 (12.5%)	0.278	0.02, 3.1	0.298			
*Other*	0	0						
Stricture Location-Distal, n, (%)	15 (83.3%)	20 (83.3%)	1	0.19, 5.15	1			
Stricture longer than 30mm, n, (%)	1 (5.6%)	10 (41.7%)	12.14	1.38, 106.7	0.024	11.8	0.66, 210	0.04
Same TUS size placed upon first TUS failure, n, (%)	3 (16.7%)	16 (66.6%)	10	2.22, 44.91	0.003	43	3.27, 565	0.004
Early first TUS failure, n, (%)	4 (22.2%)	16 (66.7%)	7	1.72, 28.33	0.006	19.2	1.59, 230	0.02

## Data Availability

The data presented in this study are available on request from the corresponding author. The data are not publicly available due topatients’ privacy.
